# One step forwards for the routine use of high‐throughput DNA sequencing in environmental monitoring. An efficient and standardizable method to maximize the detection of environmental bacteria

**DOI:** 10.1002/mbo3.421

**Published:** 2016-10-27

**Authors:** Antonia Bruno, Anna Sandionigi, Andrea Galimberti, Eleonora Siani, Massimo Labra, Clementina Cocuzza, Emanuele Ferri, Maurizio Casiraghi

**Affiliations:** ^1^ ZooPlantLabBiotechnologies and Bioscience DepartmentUniversity of Milano‐BicoccaMilanItaly; ^2^ Medicine and Surgery DepartmentUniversity of Milano‐BicoccaMonzaItaly; ^3^ FEM^2^‐AmbienteUniversity of Milano‐BicoccaMilanItaly

**Keywords:** complex matrix, DNA extraction, environmental bacteria, high‐throughput sequencing, next‐generation sequencing, qPCR, tangential flow concentration, water

## Abstract

We propose an innovative, repeatable, and reliable experimental workflow to concentrate and detect environmental bacteria in drinking water using molecular techniques. We first concentrated bacteria in water samples using tangential flow filtration and then we evaluated two methods of environmental DNA extraction. We performed tests on both artificially contaminated water samples and real drinking water samples. The efficiency of the experimental workflow was measured through qPCR. The successful applicability of the high‐throughput DNA sequencing (HTS) approach was demonstrated on drinking water samples. Our results demonstrate the feasibility of our approach in high‐throughput‐based studies, and we suggest incorporating it in monitoring strategies to have a better representation of the microbial community. In the recent years, HTS techniques have become key tools in the study of microbial communities. To make the leap from academic laboratories to the routine monitoring (e.g., water treatment plants laboratories), we here propose an experimental workflow suitable for the introduction of HTS as a standard method for detecting environmental bacteria.

## 1 Introduction

Monitoring microbial contamination in drinking water, rinse solutions, juices, milk, and many other foodstuffs is a relevant topic of public health concern (Galimberti et al., 2015; Liu et al., 2012; Namvar, Haq, Shields, Amoako, & Warriner, 2013).

The label “microbiologically pure” occurring on many food items means that no target microorganisms responsible for food spoilage and dangerous for human health (e.g., *Escherichia coli*,* Legionella pneumophila*, and Enterococci) were detected, but obviously it does not imply that there are no bacteria at all inside that product. Similarly, the European Drinking Water Directive (DWD) [Commission Directive (EU) 2015/1787 of 6 October 2015 ‐ Directory of European Union consolidated legislation] establishes the essential quality standards which water intended for human consumption must meet.

Culture‐based methods are the classical techniques applied to detect indicator or sentinel microorganisms to monitor microbial contamination (Ashbolt, Grabow, & Snozzi, 2001; Boubetra, Nestour, Allaert, & Feinberg, 2011). Nevertheless, they are biased by three relevant disadvantages: first, they require an enrichment step (typically incubation or filtration/volume reduction) that inevitably extends the analysis time. Secondly, a vast majority of bacteria are unable to grow on cultured media and are not revealed by the tests even if they occur. This discrepancy was called “the great plate count anomaly” (Hugenholtz, 2002; Staley & Konopka, 1985), and it has been well documented for several types of biological substrates. For example, in the case of bacteria inhabiting soil or aquatic environments, it is estimated that only 0.1–1% of them are able to grow on common media under standard conditions (Connon & Giovannoni, 2002; Torsvik & Øvreås, 2002; Torsvik et al., 1990). Finally, there is often the necessity of using selective media for specific bacteria, thus impeding the simultaneous detection and enumeration of different microorganisms which typically characterize complex matrices.

Molecular techniques, such as qPCR and high‐throughput DNA sequencing (HTS), may provide a way to overcome these issues (Ercolini, 2013).

The case of drinking water contaminated by an array of known and unknown microorganisms is a typical example where a complex matrix has to be monitored with standardized procedures. Classic orthogonal flow filtration (filtration on membrane disks), precipitation, centrifugation (Hill et al., 2005; Payment, Bérubé, Perreault, Armon, & Trudel, 1989; Polaczyk, Roberts, & Hill, 2007), and culture‐dependent methods are the adopted water testing method worldwide (see for instance the American Public Health Association, APHA, 2001 and the European directives, Drinking Water Directive, 98/83/EC) and were also applied in metagenomics studies to concentrate microbes from environmental samples (Cai, Yang, Jiao, & Zhang, 2015; Furtak, Dabrazhynetskaya, Volokhov, & Chizhikov, 2015; Kahler et al., 2015). Nevertheless, we are still far from gold standards. For example, orthogonal flow filtration has some relevant drawbacks too. The main limit is filter clogging when cells and other abiotic components are trapped in the filter maze. New and improved techniques are desirable to: (i) deal with heterogeneous samples showing small‐sized environmental bacteria (<0.2 μm) with low amounts of DNA and presence of inhibitors; (ii) outdo culture‐dependent techniques; (iii) increase sensitivity; (iv) decrease the response time.

Tangential flow filtration (TFF) does not rely on capturing microbes in the filter. In TFF, microorganisms and other particles remain in the bulk water samples during the filtration process, recirculating in the system. TFF has been extensively used in the biotechnology industry to recover proteins, metabolic products, plasmids, and enzymes (Van Reis & Zydney, 2001). It was only in the recent years that we registered a few TFF application to concentrate microorganisms, from endospores to viruses and pathogenic bacteria, in different liquid matrices (Cai et al., 2015; Furtak et al., 2015; Gibson & Schwab, 2011; Liu et al., 2012; Tissier, Denis, Hartemann, & Gassilloud, 2012).

Here, we propose a new laboratory workflow where TFF coupled with a high performing extraction of environmental DNA are used to overcome the biases related to the application of HTS for monitoring complex matrices. To test the effectiveness of our approach we measured, through qPCR, the recovery and sensitivity of detection in artificially contaminated water (i.e., mock community) and drinking water samples (i.e., a real case). To demonstrate that the filtration process applied in our experimental pipeline does not affect the live/dead ratio of bacteria, samples were visualized with an epifluorescence microscope before and after concentration. Finally, libraries for HTS sequencing were set up in order to demonstrate the feasibility of this pipeline in HTS‐based studies and its potential application by a wide panel of stakeholders dealing with different aims (both theoretical and applicative/commercial).

## 2 Materials and Methods

### 2.1 Bacterial strains for mock communities

We produced bacterial mock communities to artificially contaminate water samples. We used bacterial strains with different cell wall properties because these features can affect the cell lysis treatment. Therefore, we evaluated the quality and quantity of DNA extracted from monocultures and mixed cultures of gram negative and positive bacteria: *Salmonella choleraesuis* ATCC 7001, *Escherichia coli* ATCC 10536, *Pseudomonas aeruginosa* ATCC 15442, *Legionella pneumophila* ATCC 33152, *Clostridium perfringens* ATCC 13124, *Staphylococcus aureus* ATCC 6538 and *Enterococcus hirae* ATCC 10541. Furthermore, a selection of cultures (i.e., *Lactobacillus rhamnosus*,* Lactobacillus plantarum*,* Lactobacillus reuteri*,* Bifidobacterium lactis*,* Bifidobacterium longum*) was damaged with heat shock and appeared red in color after propidium iodide staining. This step allowed us to evaluate the experimental workflow even when using bacteria with compromised cell membranes.

We chose the bacteria species forming the mock community in order to maximize diversity. We included standard indicator species (i.e., *E. coli*,* C. perfringens*) and some interesting bacteria such as *Lactobacillus* spp. and *Bifidobacterium* spp., due to their recent use as a beverage additive in human health (Lee, Boo, & Liu, 2013) and water additive in aquaculture (Dash et al., 2016).

The species tested were strains cultivated at the Department of Biotechnologies and Biosciences of the University of Milano‐Bicocca, Italy.

Serial 10‐fold dilutions were prepared and CFU of each live bacteria were estimated by plating on specific media.

Optical densities (OD_600_) and/or CFU of each monoculture are listed in Table S1. Only optical densities were reported for damaged cultures.

### 2.2 Tangential flow filtration

The TFF system involved a peristaltic pump (Masterflex L/S Economy Drive), Tygon^®^ tubing, sterile reservoirs and filtration modules. The tangential flow filter used was a VivaFlow 200 cassette [Sartorius Italy S.r.l., Muggiò (MB), Italy] made of polyethersulfone (PES) with a nominal pore rating of 10000 MWCO and a surface area of 200 cm^2^. The system was scaled up with an additional unit connected in parallel to increase the filtration surface area and the flow speed.

All tubing, tubing connections, and containers were sterilized with sodium hypochlorite or autoclaved prior to each experiment. Every step was conducted in the laminar flow cabinet. The TFF system was run at a transmembrane pressure of 1.5 bar.

The initial water samples were concentrated to a final retentate volume of 100 ml. Three aliquots of filtrate (that should not contain bacteria) were conserved to verify the absence of bacteria/DNA.

### 2.3 Live/dead visualization

All bacterial monocultures, the prefiltration mix of bacteria, the spiked water, the concentrated water, and the filtrated were visualized at an epifluorescent microscope (Nikon Y‐FL) at 600× magnification.

A quantity of 20 μl of each sample was stained with 20 μl of 2× solution of SYTO9/propidium iodide (BacLight Bacterial Viability kit, Thermo Fisher Scientific, Milan, Italy) and incubated in the dark, at 4°C, for 15 min.

SYTO 9 is a dye with similar properties of SYBR GREEN I, allowing live cell staining. Otherwise, propidium iodide penetrates only damaged cell membrane, quenching SYTO 9 fluorescence and giving red coloration to the cells. The excitation/emission wavelength is 480/500 nm for SYTO 9 stain and 490/635 nm for propidium iodide.

The live/dead ratio was estimated, with particular attention paid for preconcentration and postconcentration samples [(Patel et al., 2007) with minor modifications].

All counts were made in triplicate.

### 2.4 Intra‐assay repeatability tests

**Figure 1:**
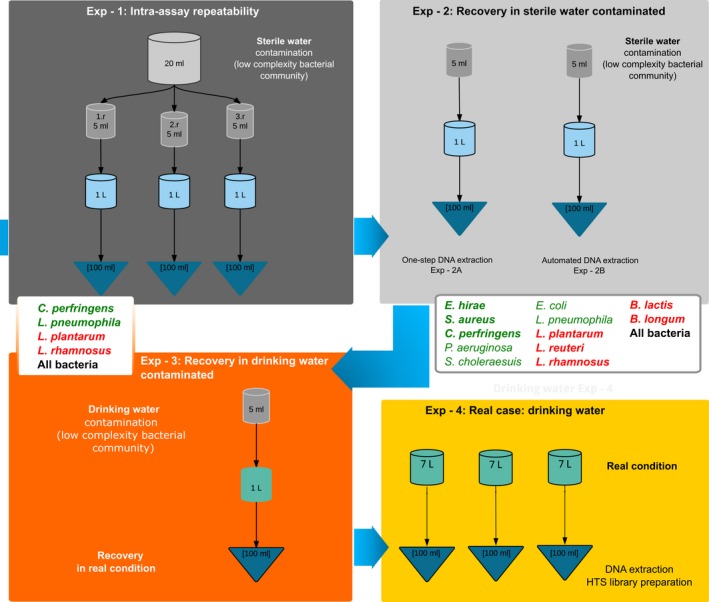
Experimental workflow to test and validate the efficacy of TFF. In the Exp ‐ 1 box, reproducibility was tested (see Section 2.4). The panels Exp ‐ 2A and Exp ‐ 2B report the artificial contamination experiment comparing two different types of DNA extraction (A and B, see Section 2.5). The Exp ‐ 3 panel illustrates the experiment using drinking water samples, instead of artificially contaminated sterile water (see Section 2.5). Finally, the Exp ‐ 4 panel represents the experiment in real conditions using drinking water samples, without artificial contamination (see Section 2.6). Gray cylinders represent the contamination solutions, blue cylinders represent “pre” concentration samples, and dark blue triangles represent “post” concentration samples. Bacteria used for each experiment are listed in white boxes: in green live bacteria, in red dead bacteria, in bold gram‐positive bacteria, and normal font refers to gram‐negative bacteria

In the first experiment, artificially contaminated samples were prepared in order to test the intra‐assay repeatability of TFF and DNA extraction (Figure 1, Exp ‐ 1).

Four different bacterial species, two alive and two dead (randomly chosen, listed in Table S2), were used to contaminate 20 ml of Milli‐Q sterile water. With 5 ml of this contamination mix, three identically spiked 1‐liter samples were created to estimate intra‐assay repeatability.

Genomic DNA (gDNA) extraction was carried out on the samples before and after the concentration process and from filtrate. Three replicates of one‐step rapid DNA extraction with Instagene Matrix [Bio‐Rad S.r.l., Segrate (MI), Italy] were performed. The Instagene procedure utilizes a lysis solution (Chelex^®^). Manufacture instructions were followed: 200 μl of lysis solution were added to 200 μl of sample and this mixture was then incubated at 56°C and 94°C. A volume of 200 μl of supernatant containing DNA was used for the tests or stored at −80 °C.

### 2.5 Recovery tests

In the second experiment, bacteria recovery efficiency was first tested using artificially contaminated sterile water. The number of bacterial species used was increased to 12, both dead and alive, as listed in Table S2. One liter of spiked sterile water was concentrated using TFF. DNA extraction was carried out as described in Section 2.4 (Figure 1, Exp ‐ 2A).

Moreover, a second DNA extraction method was included in the analysis to test an automated system, using NucliSens^®^ EasyMAG^™^ (Biomerieux Italia S.p.a., Florence, Italy) (Figure 1, Exp ‐ 2B; Table S10). The specific protocol for increasing the DNA yield was used. The nucleic acids were eluted in a final volume of 50 μl of elution buffer and stored at −80°C for further tests.

In the third experiment, 1 L of drinking water (Table S3) was artificially contaminated with the bacterial mix described in Table S2 and concentrated to 50 ml, to evaluate the effect of environmental samples characteristics on the method. One‐step DNA extraction (Bio‐Rad) was used (see Section 2.4) (Figure 1, Exp ‐ 3).

### 2.6 Environmental (drinking water) samples

Three samples of drinking water (7 L each, Table S3) from a drinking water treatment plant in Milan (managed by Metropolitana Milanese S.p.A.) were tested, to verify the applicability in the case of environmental samples characterized by low bacterial concentration (Bruno A., Sandionigi A., Bernasconi M., Labra M., Casiraghi M. in prep). Nucleic acid extraction was performed with both the methods, the one‐step DNA extraction (Bio‐Rad) and the automated DNA extraction (Biomerieux) (Figure 1, Exp ‐ 4).

### 2.7 qPCR

Quantitative real‐time PCR (qPCR) assays were performed with AB 7500 (Applied Biosystem).

Samples before the concentration process (called “PRE”) and after (called “POST”) were tested. Dilutions were used.

qPCR conditions included an initial denaturation at 95°C for 10 min, followed by 40 cycles of denaturation at 95°C for 15 s and annealing‐elongation for 1 min. Real‐time PCR was set up with 2X SsoFast EvaGreen Supermix with Low ROX [Bio‐Rad S.r.l., Segrate (MI), Italy] in which EvaGreen was used as a detecting dye; a 10 μl reaction consisted of 5.0 μl SsoFast EvaGreen Supermix with Low ROX, 0.1 μl each 10 μmol/L primer solution, 2 μl DNA sample, and 2.8 μl of Milli‐Q water. Primer sequences, targets, annealing temperatures, and references are given in Table S4.

Standard curves were generated using 10‐fold serial dilutions of positive controls and qPCR amplification efficiencies (*E*) were based on the following Equation (1):

(1) $$E=10^{\left(-1/\text{slope}\right)}-1,$$

and *R* ^2^ values (linearity) were 0.99.

All samples and standards were run in triplicate.

Negative controls were also tested in triplicate too for each amplification. All the assays were followed by a dissociation stage and melting curves were obtained.

Amplification data were collected and analyzed with the SDS 7500 Real‐Time PCR System Software (Applied Biosystems).

### 2.8 Recovery efficiencies and qPCR statistical analysis

We reported all the data derived from qPCR as DNA copies of the target amplified rather than as CFUs, to better estimate dead and viable but nonculturable cells.

In order to apply a generalized linear mixed model (GLMM) under Poisson‐lognormal error, to account for higher variation at the lower end of target abundance and calculate the recovery rates, MCMC.qpcr R package (Matz, et al. 2013) was used to convert Ct (Threshold Cycles) data in bacterial counts. Geometric means were calculated.

The conversion to approximate counts uses the formula:

(2) Count=E(Ct1 ‐ Ct),

where *E* is the efficiency of amplification and Ct1 is the number of qPCR cycles required to detect a single target molecule.

To estimate the efficiency of the method, we decided to use recovery efficiencies, representing the rate of targets detected after concentration and DNA extraction processes, thus estimating the efficiency of the method.

Recovery efficiencies (*R*) were calculated using the equation

(3) R={[counts(f)/counts(i)]∗100}/F

where counts(*f*) is the counts value corresponding to the quantity of DNA copies extracted after concentration, counts(*i*) before concentration and *F* is the factor of concentration. Values were expressed as percentages.

To determine whether TFF recovery efficiency varied among the tested targets, a one‐way analysis of variance ANOVA in combination with Tukey post hoc tests was used to find significant differences between the measured means of recovery efficiency. A probability of *p *<* *.05 was considered to indicate a significant difference.

The relationships between recovery efficiency, microbial abundance, and experimental factors were investigated using generalized linear mixed models (GLMM).

The Markov chain Monte Carlo (MCMC) algorithm implemented in the package is used to sample from the joint posterior distribution over all model parameters in order to estimate the effects of all experimental factors on the levels of the specific microbial species.

Generalized linear mixed models used to test the effect of different experimental conditions and their results were reported in the R Markdown report in Supplementary information as a table, where the bacteria abundances are listed for each bacterium. Results were plotted using ggplot2 R package (Wickham, 2009).

### 2.9 Library preparation for Illumina MiSeq sequencing

To test the protocols in a “real case”, the three samples of drinking water described in Exp ‐ 4 were sequenced using high‐throughput DNA sequencing techniques. These samples were chosen randomly from a wider water monitoring study (Bruno et al. in prep), where a total of 42 samples of water microbiome were sequenced in the same run. Illumina MiSeq 16S (V3–V4 regions of 16S rRNA gene) libraries were generated following standard protocol (16S Metagenomic Sequencing Library Preparation, Part # 15044223 Rev. B) with modifications, due to the low DNA concentrations. Specifically, DNA extracts were normalized on Ct values of qPCR with the same primer pairs, instead of measuring the total amount of microbial DNA with fluorometric/spectrophotometric methods.

Amplicon PCR was performed using the primer pairs: 5′TCGTCGGCAGCGTCAGATGTGTATAAGAGACAGCCTACGGGNGGCWGCAG3′ 5′GTCTCGTGGGCTCGGAGATGTGTATAAGAGACAGGACTACHVGGGTATCTAATCC3′ at an initial concentration of [10 μmol/L], with the aim of increasing the volume of DNA in the reaction.

The PCR‐clean up step after amplicon PCR was modified in the final resuspension volume, with a twofold increase of sample concentration.

Libraries were quantified with 2100 Bioanalyzer (Agilent Technologies) and samples were sequenced using the 2 × 300 paired‐end chemistry (MiSeq Reagent Kit v3). The three samples were sequenced in the same run together with other 39 other samples. In order to verify the sequencing reproducibility, the technical replicates of each sample were sequenced in a second run, in the same conditions.

### 2.10 Sequence analysis

Illumina reads were paired and preprocessed using the USEARCH script (Edgar, 2010). During the quality filter step, reads were filtered out if: (i) ambiguous bases were detected, (ii) reads lengths were outside the bounds of 250 bp and/or (iii) the average quality scores over a sliding window of 40 bp dropped below a value of 25. Reads were then processed with VSEARCH v. 1.1.3 software (https://github.com/torognes/vsearch), which removed noise and chimeras prior to performing de novo clustering into OTUs at 100% sequence identity (i.e., the amount of characters which match exactly between two different sequences) and discarding those clusters encompassing <100 sequences.

In order to estimate the sequences' diversity, the number of obtained clusters (OTUs) was calculated for each sample. Shared OTUs, present for more than 0.1% when considering the all observations (sequences), were calculated with *shared_phylotypes.py* script of QIIME (Caporaso et al., 2010) suite tools.

### 2.11 Accession number(s)

Sequencing data were deposited in the National Center for Biotechnology Information (NCBI) Sequence Read Archive (SRA) under accession no. SAMN04364347, SAMN04364450, SAMN04364365, SAMN04364389, SAMN04364392, SAMN04364407.

## 3 Results

### 3.1 qPCR efficiency

qPCR standards were analyzed in order to determine the reactions efficiency. The slope of the standards ranged from −3.8 to −3.26. The amplification efficiency values ranged from 83% to 103%, and the correlation coefficient (*R* ^2^) ranged from 0.98 to 0.99.

### 3.2 Intra‐assay repeatability in artificially contaminated water samples

**Figure 2:**
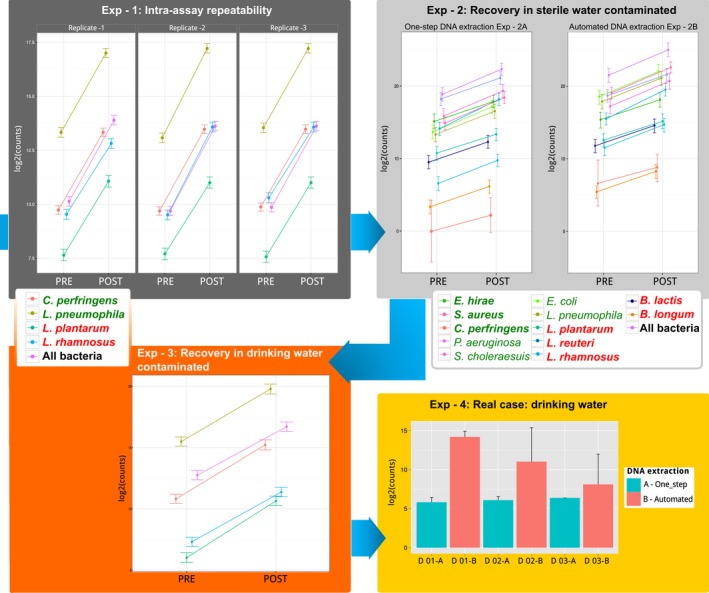
Results obtained from the experiments illustrated in Figure 1. On the y axis, the log_2_ (DNA copies) is reported. On the *x* axis, measures obtained from samples before filtration (PRE) and after filtration (POST) are reported. Bacteria used to contaminate water samples are listed in white boxes: in green live bacteria, in red dead bacteria, in bold gram‐positive bacteria, and normal font refers to gram‐negative bacteria. In the case of the experiment with drinking water (Exp ‐ 4) only data after‐filtration are reported

In the first experiment (Figure 1, Exp ‐ 1 panel; Figure 2, Exp ‐ 1 panel), intra‐assay repeatability was estimated in terms of counts, for each target, as described in Section 2.8. The three identical spiked samples showed no significant differences across all the replicates of the pre‐ and postconcentration samples for each target (*p *>* *.05), demonstrating the repeatability of the procedure, from the filtration to the DNA extraction. The only exception was represented by *L. rhamnosus,* including both pre‐ and postconcentration samples (Figure 2, Table S5).

### 3.3 Recovery in artificially contaminated water samples

In the second experiment (Figure 1, Exp ‐ 2A panel; Figure 2, Exp ‐ 2A panel), we used a bacterial mix with a higher complexity, which increased the number of species tested to 12, and recovery efficiencies were calculated. All the targets were detected after the concentration process. The recovery efficiency was greater than 61% in all cases, and there was always a statistically significant difference (*p *<* *.05) between samples before and after concentration, for each target (Table S6), when considering the counts (as described in section 2.8). No significant difference existed in the recoveries between gram‐positive and gram‐negative bacteria (*p *>* *.05). Moreover, no significant difference in recoveries was found between live and dead bacteria (*p *>* *.05). The only exception was represented by *C. perfringens*, which was not detected in the samples before the concentration process, but was detected after concentration. For this reason, the estimation of the recovery efficiency was not possible in this case.

In addition to the one‐step DNA extraction method, in this experiment, we tested the automated extraction method on the same samples (Figure 2, Exp ‐ 2B panel), showing that count values in general were not significantly higher than those obtained with one‐step DNA extraction (see GLMM model results in SI). Noticeably, *C. perfringens* was detected even in the sample before concentration, with a recovery of 49% (Table S5).

To verify the feasibility and efficacy of our approach in real conditions, environmental (drinking water) samples (Table S6) were artificially contaminated with the mix described in Table S2 in Exp ‐ 3 and in Figure 1, Exp ‐ 3 panel.

After the TFF step, all the bacteria were successfully recovered in artificially contaminated environmental samples.

Our results showed that the recovery efficiencies were always >77%, even in the case of environmental samples, that can be characterized by the presence of inhibitors of amplification. No significant differences existed in the recoveries between gram‐positive and gram‐negative bacteria as well as between live and dead bacteria (*p *>* *.05), suggesting that a damaged cell membrane can fully stand the pressure exercised by TFF (Table S6).

### 3.4 Recovery in drinking water samples

When three samples of drinking water from the water treatment plant were additionally tested (Figure 1, Exp ‐ 4 panel), we verified the applicability of our approach with environmental samples (Figure 2, Exp ‐ 4 panel). It was not possible to detect DNA molecules in the samples before tangential flow concentration. After TFF, environmental bacteria DNA was observed in all the samples. Automated DNA extraction was significantly more efficient than one‐step DNA extraction when considering count values (ANOVA: *p *<* *.05) (Table S7).

### 3.5 Library preparation and high‐throughput DNA sequencing

Libraries generated with DNA extracts derived from one‐step lysis gave no results after quantification due to the low amount of starting DNA. For this reason, they were not considered for sequencing.

Libraries were successfully generated from drinking water DNA extracts obtained with the automated system. DNA concentration after library preparation was proportional to the number of reads obtained in HTS, performed with the same primer pairs. Reads obtained from drinking water samples concentrated and extracted with the automated system ranged from 4478–7199 (sample D‐02) to 43121–63904 (sample D‐01) (Table S8). The observed OTUs ranged from 692–971 (sample D‐02) to 2780–2882 (sample D‐01).

The sample with the highest yield of reads also showed the highest number of OTUs. The two sequencing replicates for each samples turned out to be very similar in terms of the number of reads and observed OTUs. Each replicate shared more than 98% of more frequent OTUs (more than 0.01% of the total observations) (Table S8).

### 3.6 Live/dead ratio variations

Since the presence of microbial DNA is not a direct measure of viable organisms (Jofre & Blanch, 2010; Nocker, Richter‐Heitmann, Montijn, Schuren, & Kort, 2010), we decided to partially overcome this disadvantage using microscopy visualization after live/dead staining. In this way, it was possible to obtain an overall estimation of the live/dead ratio.

Single‐species cultures were checked at the epifluorescence microscope and live/dead ratios were reported in Table S9.

The live/dead ratio was estimated for samples from spiked solutions and after the concentration process. No differences were shown in the bacteria viability proportion after the concentration process. Moreover, damaged cell membranes can stand the pressure exercised by the peristaltic pump, as we noticed in the samples after TFF. No cells were detected in filtrate, for each sample tested.

## 4 Discussion

Our results demonstrate that tangential flow filtration (TFF) coupled with DNA‐based molecular techniques is the ideal tool for surveying microbial diversity in water samples and allows an unbiased and sensitive detection of microbes. The first advantage is in terms of analysis time. In our study, we were able to concentrate 1 L of drinking water in <15 min at a pressure of 1 bar using our TFF system with a nominal pore rating of 10000 MWCO. To give a comparison, the orthogonal filtration (with a vacuum pump Vacuubrand, 2.0/2.2 m^3^/h) of 100 ml of the same drinking water sample that we tested in our laboratory took 10 min, with less stringent filtration conditions (filter membrane with pore size of 0.1 μm). The striking urgency for reliable protocols to concentrate the widest spectrum of bacterial diversity using more stringent conditions is due to the small size of some environmental bacteria recently described in groundwater (Kempes, Wang, Amend, Doyle, & Hoehler, 2016; Koch, 1996; Luef et al., 2015; Size Limits of Very Small Microorganisms, 1999).

Secondly, we confirmed the intra‐assay repeatability of the method. The filtration process was efficient in concentrating microorganisms and did not affect their viability, as demonstrated through qPCR combined with microscopy visualization.

Our results also support the importance of choosing the right DNA extraction method. Even if no significant difference for most of the targets was measured between the two DNA extraction approaches (i.e., the one‐step lysis and the automated system), one of the targets was noticeably detected before the concentration process using automated system, but not using one‐step extraction. Both DNA extraction methods were rapid and had a reduced contamination risk with exogenous DNA. Nevertheless, the automated system was found to significantly increase the yield of DNA obtained due to a higher sensitivity, magnetic beads, and better removal of PCR inhibitors.

For these reasons, we suggest the use of TFF coupled with one‐step DNA extraction when an accurate quantitative detection of target microorganisms/genes is required to take advantage of qPCR sensitivity. Conversely, when a qualitative analyses of the entire microbial community is the aim of the research, a HTS approach is critical. In this case, it is necessary to use a more sensitive DNA extraction method, such the automated system based on magnetic beads.

Concerning the HTS characterization of the water microbiome, our modifications to the library protocol to increase DNA concentration proved to be crucial for successful sequencing. 16S rDNA massive sequencing generated the expected number of reads, characterized by a high diversity (expressed as unique sequences). These results are in agreement with recent scientific studies regarding drinking water, reporting that drinking water microbial communities are complex, comprising up to 48 phyla and in excess of 4 000 unique operational taxonomical units (OTUs) (Pinto, Xi, & Raskin, 2012; Proctor & Hammes, 2015). This was verified for each sample and for both the sequencing replicates, confirming that the modified protocol for library preparation did not affect the sequencing process.

On the whole, the obtained results highlighted that the experimental workflow proposed here is flexible and adaptable even under real conditions where the presence of inhibitors can affect recovery. Indeed, even in the experiment where we used artificially contaminated drinking water (instead of Milli‐Q water), we achieved appreciable recoveries.

Our data strongly supports the use of HTS in monitoring strategies, even outside academic laboratories, and in all situations in which routine and standard protocols are required. Microbial communities result in a continuum of genetic diversity that greatly complicates the identification of closely related microbial taxa. Although imperfect, the introduction of OTU concept, mostly based on small subunit ribosomal RNA gene similarities, has offered several new insights into microbial ecology studies (Zinger, Gobet, & Pommier, 2012). New solutions are needed in a field where widespread microdetection can find new targets that can affect human health. This need for new laboratory protocols is crucial, for example, in antibiotic resistance studies which is an emerging topic in waters for human consumption (Alexander, Bollmann, Seitz, & Schwartz, 2015; Berendonk et al., 2015).Overall, this work highlights the complexity and the importance of correctly addressing a biological question and choosing the most appropriate tools to get closest to the answer/s.

## Conflict of Interest

No conflict of interest declared.
