# Supplementary material for: One step forwards for the routine use of high‐throughput DNA sequencing in environmental monitoring. An efficient and standardizable method to maximize the detection of environmental bacteria
Source: Microbiologyopen. 2016 Oct 27;6(1):e00421. doi: 10.1002/mbo3.421 (PMC5300880; doi:10.1002/mbo3.421)
Supplement: Supplementary file 1 [file MBO3-6-0-s001.pdf]

result_mcmc_qpcr

Anna Sandionigi & Antonia Bruno

28 giugno 2016

```
knitr::opts_chunk$set(echo = TRUE)
```

```
library("MCMC.qpcr")
```

```
## Loading required package: MCMCglmm

## Loading required package: Matrix

## Loading required package: coda

## Loading required package: ape

## Loading required package: ggplot2
```

```
library("MCMCglmm")
library("ggplot2")
```

```
setwd("/home/anna/Scrivania/shared_jobs/progetto_acqua/paper_protocol_water/Sup_materials/")
```

Import data in MCMC.qpcr package

```
eff <- read.csv("dataset_all_species.csv")
###calculate efficens
c.eff <- read.csv("c.eff.csv")
##trasform count with mcmc
qs_mock= cq2counts(data=eff, effic=c.eff, genecols=c(7:19), condcols=c(1:6))
```

We regenerate csv separatelly because subsampling can generate missing values in random variable "sample" Files needed for the downstream analysis are provided in Supplementary files

Experiment 1 ((fig 1 -A)

Test reproducibility in Milli-Q water with bacterial contaminants Species involved = Legionella pneumophila, Chlostridium perfringens , Lactobacillus rhamnosus, Lactobacillus plantarum and panbacterial marker

```
exp1.d <- read.csv("exp_1.csv")
```

```
exp1.m= mcmc.qpcr(
    fixed="pre_post+repl_conc",
    burnin=4000,
    data= exp1.d)
```

```r
s1$summary
```

```
##       gene pre_post repl_conc      mean        sd     lower     upper
## 1  CLOSTRI    1.pre       1.r  9.736060 0.1186544  9.534151  9.933622
## 2  CLOSTRI    1.pre       2.r  9.687483 0.1252822  9.474173  9.889552
## 3  CLOSTRI    1.pre       3.r  9.879902 0.1217920  9.675577 10.073357
## 4  CLOSTRI   2.post       1.r 13.336421 0.1251430 13.123651 13.540235
## 5  CLOSTRI   2.post       2.r 13.480263 0.1201343 13.288313 13.663558
## 6  CLOSTRI   2.post       3.r 13.480263 0.1201343 13.288313 13.663558
## 7    LEGIO    1.pre       1.r 13.328800 0.1345593 13.114750 13.540966
## 8    LEGIO    1.pre       2.r 13.083675 0.1325296 12.876352 13.302482
## 9    LEGIO    1.pre       3.r 13.549441 0.1283901 13.342816 13.766294
## 10   LEGIO   2.post       1.r 16.990148 0.1340400 16.767769 17.215505
## 11   LEGIO   2.post       2.r 17.210789 0.1405726 16.979280 17.444650
## 12   LEGIO   2.post       3.r 17.210789 0.1405726 16.979280 17.444650
## 13  LPLANT    1.pre       1.r  7.636045 0.1631191  7.364426  7.904259
## 14  LPLANT    1.pre       2.r  7.705401 0.1601253  7.443307  7.959601
## 15  LPLANT    1.pre       3.r  7.567710 0.1584249  7.307931  7.830160
## 16  LPLANT   2.post       1.r 11.072532 0.1541161 10.831720 11.327684
## 17  LPLANT   2.post       2.r 11.004197 0.1599402 10.739708 11.259745
## 18  LPLANT   2.post       3.r 11.004197 0.1599402 10.739708 11.259745
## 19   LRHAM    1.pre       1.r  9.532099 0.1309329  9.312110  9.743419
## 20   LRHAM    1.pre       2.r  9.499593 0.1363735  9.267972  9.716728
## 21   LRHAM    1.pre       3.r 10.294900 0.1393277 10.070624 10.526462
## 22   LRHAM   2.post       1.r 12.823916 0.1348803 12.597010 13.030272
## 23   LRHAM   2.post       2.r 13.586717 0.1332234 13.375744 13.814822
## 24   LRHAM   2.post       3.r 13.586717 0.1332234 13.375744 13.814822
## 25 PANBACT    1.pre       1.r 10.136946 0.1274500  9.916428 10.343320
## 26 PANBACT    1.pre       2.r  9.694322 0.1310607  9.477161  9.910010
## 27 PANBACT    1.pre       3.r  9.850607 0.1283678  9.644443 10.056454
## 28 PANBACT   2.post       1.r 13.890890 0.1318793 13.680350 14.105547
## 29 PANBACT   2.post       2.r 13.604550 0.1327355 13.389661 13.821961
## 30 PANBACT   2.post       3.r 13.604550 0.1327355 13.389661 13.821961
```

```r
summary(exp1.m)
```

```
##
##  Iterations = 4001:12991
##  Thinning interval  = 10
##  Sample size  = 900
##
##  DIC: 3248.781
##
##  G-structure:  ~sample
##
##        post.mean  l-95% CI  u-95% CI eff.samp
## sample 4.745e-10 1.811e-47 2.075e-11        0
##
##  R-structure:  ~idh(gene):units
##
##                    post.mean l-95% CI u-95% CI eff.samp
## geneCLOSTRI.units   0.09126  0.05865   0.1249    900.0
```

```
## geneLEGIO.units     0.11679  0.07805   0.1640    802.7
## geneLPLANT.units    0.16097  0.10287   0.2266    900.0
## geneLRHAM.units     0.12008  0.07409   0.1610    900.0
## genePANBACT.units   0.11003  0.06758   0.1543   1030.8
##
##  Location effects: count ~ 0 + gene + +gene:pre_post + gene:repl_conc
##
##                          post.mean l-95% CI u-95% CI eff.samp   pMCMC
## geneCLOSTRI                6.74852  6.59053  6.91881      900 < 0.001 **
## geneLEGIO                  9.23882  9.05613  9.41278      900 < 0.001 **
## geneLPLANT                 5.29290  5.07731  5.51612     1128 < 0.001 **
## geneLRHAM                  6.60715  6.42779  6.77730      900 < 0.001 **
## genePANBACT                7.02640  6.83568  7.17569      900 < 0.001 **
## geneCLOSTRI:pre_post2.post   2.49558  2.33446  2.67353      900 < 0.001 **
## geneLEGIO:pre_post2.post     2.53785  2.34337  2.70178      900 < 0.001 **
## geneLPLANT:pre_post2.post    2.38199  2.18419  2.60567      900 < 0.001 **
## geneLRHAM:pre_post2.post     2.28171  2.09824  2.46845      900 < 0.001 **
## genePANBACT:pre_post2.post   2.60204  2.41318  2.76854      900 < 0.001 **
## geneCLOSTRI:repl_conc2.r    -0.03367 -0.23810  0.17291      900 0.74000
## geneLEGIO:repl_conc2.r      -0.16991 -0.40120  0.03960      900 0.12444
## geneLPLANT:repl_conc2.r      0.04807 -0.22240  0.30664      900 0.71556
## geneLRHAM:repl_conc2.r      -0.02253 -0.24676  0.19945     1100 0.84000
## genePANBACT:repl_conc2.r    -0.30680 -0.51750 -0.11051      900 0.00889 **
## geneCLOSTRI:repl_conc3.r     0.09970 -0.12841  0.28006      900 0.34444
## geneLEGIO:repl_conc3.r       0.15294 -0.05551  0.39846      900 0.17111
## geneLPLANT:repl_conc3.r     -0.04737 -0.32956  0.19013      900 0.70000
## geneLRHAM:repl_conc3.r       0.52873  0.31157  0.75063     1112 < 0.001 **
## genePANBACT:repl_conc3.r    -0.19848 -0.41515  0.01585      900 0.07111 .
## ---
## Signif. codes:  0 '***' 0.001 '**' 0.01 '*' 0.05 '.' 0.1 ' ' 1
```

```
s1$geneWise
```

```
## $CLOSTRI
##                              difference
## pvalue                        pre_post1.pre:repl_conc1.r
##   pre_post1.pre:repl_conc1.r                          NA
##   pre_post1.pre:repl_conc2.r                   0.7470620
##   pre_post1.pre:repl_conc3.r                   0.3355047
##   pre_post2.post:repl_conc1.r                  0.0000000
##   pre_post2.post:repl_conc2.r                  0.0000000
##   pre_post2.post:repl_conc3.r                  0.0000000
##                              difference
## pvalue                        pre_post1.pre:repl_conc2.r
##   pre_post1.pre:repl_conc1.r                 -0.04857696
##   pre_post1.pre:repl_conc2.r                          NA
##   pre_post1.pre:repl_conc3.r                  0.19298214
##   pre_post2.post:repl_conc1.r                 0.00000000
##   pre_post2.post:repl_conc2.r                 0.00000000
##   pre_post2.post:repl_conc3.r                 0.00000000
##                              difference
## pvalue                        pre_post1.pre:repl_conc3.r
##   pre_post1.pre:repl_conc1.r                   0.1438418
##   pre_post1.pre:repl_conc2.r                   0.1924188
```

```
##   pre_post1.pre:repl_conc3.r                        NA
##   pre_post2.post:repl_conc1.r                0.0000000
##   pre_post2.post:repl_conc2.r                0.0000000
##   pre_post2.post:repl_conc3.r                0.0000000
##                              difference
## pvalue                        pre_post2.post:repl_conc1.r
##   pre_post1.pre:repl_conc1.r                  3.6003607
##   pre_post1.pre:repl_conc2.r                  3.6489377
##   pre_post1.pre:repl_conc3.r                  3.4565189
##   pre_post2.post:repl_conc1.r                        NA
##   pre_post2.post:repl_conc2.r                 0.3355047
##   pre_post2.post:repl_conc3.r                 0.3355047
##                              difference
## pvalue                        pre_post2.post:repl_conc2.r
##   pre_post1.pre:repl_conc1.r                  3.7442025
##   pre_post1.pre:repl_conc2.r                  3.7927795
##   pre_post1.pre:repl_conc3.r                  3.6003607
##   pre_post2.post:repl_conc1.r                 0.1438418
##   pre_post2.post:repl_conc2.r                        NA
##   pre_post2.post:repl_conc3.r                       NaN
##                              difference
## pvalue                        pre_post2.post:repl_conc3.r
##   pre_post1.pre:repl_conc1.r                  3.7442025
##   pre_post1.pre:repl_conc2.r                  3.7927795
##   pre_post1.pre:repl_conc3.r                  3.6003607
##   pre_post2.post:repl_conc1.r                 0.1438418
##   pre_post2.post:repl_conc2.r                 0.0000000
##   pre_post2.post:repl_conc3.r                        NA
##
## $LEGIO
##                              difference
## pvalue                        pre_post1.pre:repl_conc1.r
##   pre_post1.pre:repl_conc1.r                        NA
##   pre_post1.pre:repl_conc2.r                 0.1281797
##   pre_post1.pre:repl_conc3.r                 0.1820303
##   pre_post2.post:repl_conc1.r                0.0000000
##   pre_post2.post:repl_conc2.r                0.0000000
##   pre_post2.post:repl_conc3.r                0.0000000
##                              difference
## pvalue                        pre_post1.pre:repl_conc2.r
##   pre_post1.pre:repl_conc1.r              -0.245125432
##   pre_post1.pre:repl_conc2.r                        NA
##   pre_post1.pre:repl_conc3.r               0.004464364
##   pre_post2.post:repl_conc1.r              0.000000000
##   pre_post2.post:repl_conc2.r              0.000000000
##   pre_post2.post:repl_conc3.r              0.000000000
##                              difference
## pvalue                        pre_post1.pre:repl_conc3.r
##   pre_post1.pre:repl_conc1.r                 0.2206410
##   pre_post1.pre:repl_conc2.r                 0.4657664
##   pre_post1.pre:repl_conc3.r                        NA
##   pre_post2.post:repl_conc1.r                0.0000000
##   pre_post2.post:repl_conc2.r                0.0000000
##   pre_post2.post:repl_conc3.r                0.0000000
```

```
##                              difference
## pvalue                        pre_post2.post:repl_conc1.r
##   pre_post1.pre:repl_conc1.r                    3.6613474
##   pre_post1.pre:repl_conc2.r                    3.9064728
##   pre_post1.pre:repl_conc3.r                    3.4407064
##   pre_post2.post:repl_conc1.r                          NA
##   pre_post2.post:repl_conc2.r                   0.1820303
##   pre_post2.post:repl_conc3.r                   0.1820303
##                              difference
## pvalue                        pre_post2.post:repl_conc2.r
##   pre_post1.pre:repl_conc1.r                     3.881988
##   pre_post1.pre:repl_conc2.r                     4.127114
##   pre_post1.pre:repl_conc3.r                     3.661347
##   pre_post2.post:repl_conc1.r                    0.220641
##   pre_post2.post:repl_conc2.r                          NA
##   pre_post2.post:repl_conc3.r                         NaN
##                              difference
## pvalue                        pre_post2.post:repl_conc3.r
##   pre_post1.pre:repl_conc1.r                     3.881988
##   pre_post1.pre:repl_conc2.r                     4.127114
##   pre_post1.pre:repl_conc3.r                     3.661347
##   pre_post2.post:repl_conc1.r                    0.220641
##   pre_post2.post:repl_conc2.r                    0.000000
##   pre_post2.post:repl_conc3.r                          NA
##
## $LPLANT
##                              difference
## pvalue                        pre_post1.pre:repl_conc1.r
##   pre_post1.pre:repl_conc1.r                         NA
##   pre_post1.pre:repl_conc2.r                  0.7256939
##   pre_post1.pre:repl_conc3.r                  0.7221393
##   pre_post2.post:repl_conc1.r                 0.0000000
##   pre_post2.post:repl_conc2.r                 0.0000000
##   pre_post2.post:repl_conc3.r                 0.0000000
##                              difference
## pvalue                        pre_post1.pre:repl_conc2.r
##   pre_post1.pre:repl_conc1.r                 0.06935582
##   pre_post1.pre:repl_conc2.r                         NA
##   pre_post1.pre:repl_conc3.r                 0.48520144
##   pre_post2.post:repl_conc1.r                0.00000000
##   pre_post2.post:repl_conc2.r                0.00000000
##   pre_post2.post:repl_conc3.r                0.00000000
##                              difference
## pvalue                        pre_post1.pre:repl_conc3.r
##   pre_post1.pre:repl_conc1.r                -0.06833548
##   pre_post1.pre:repl_conc2.r                -0.13769130
##   pre_post1.pre:repl_conc3.r                         NA
##   pre_post2.post:repl_conc1.r                0.00000000
##   pre_post2.post:repl_conc2.r                0.00000000
##   pre_post2.post:repl_conc3.r                0.00000000
##                              difference
## pvalue                        pre_post2.post:repl_conc1.r
##   pre_post1.pre:repl_conc1.r                    3.4364872
##   pre_post1.pre:repl_conc2.r                    3.3671314
```

```
##   pre_post1.pre:repl_conc3.r                    3.5048227
##   pre_post2.post:repl_conc1.r                          NA
##   pre_post2.post:repl_conc2.r                   0.7221393
##   pre_post2.post:repl_conc3.r                   0.7221393
##                              difference
## pvalue                        pre_post2.post:repl_conc2.r
##   pre_post1.pre:repl_conc1.r                   3.36815171
##   pre_post1.pre:repl_conc2.r                   3.29879590
##   pre_post1.pre:repl_conc3.r                   3.43648719
##   pre_post2.post:repl_conc1.r                 -0.06833548
##   pre_post2.post:repl_conc2.r                          NA
##   pre_post2.post:repl_conc3.r                         NaN
##                              difference
## pvalue                        pre_post2.post:repl_conc3.r
##   pre_post1.pre:repl_conc1.r                   3.36815171
##   pre_post1.pre:repl_conc2.r                   3.29879590
##   pre_post1.pre:repl_conc3.r                   3.43648719
##   pre_post2.post:repl_conc1.r                 -0.06833548
##   pre_post2.post:repl_conc2.r                  0.00000000
##   pre_post2.post:repl_conc3.r                          NA
##
## $LRHAM
##                              difference
## pvalue                        pre_post1.pre:repl_conc1.r
##   pre_post1.pre:repl_conc1.r                         NA
##   pre_post1.pre:repl_conc2.r               8.451038e-01
##   pre_post1.pre:repl_conc3.r               1.674617e-06
##   pre_post2.post:repl_conc1.r              0.000000e+00
##   pre_post2.post:repl_conc2.r              0.000000e+00
##   pre_post2.post:repl_conc3.r              0.000000e+00
##                              difference
## pvalue                        pre_post1.pre:repl_conc2.r
##   pre_post1.pre:repl_conc1.r              -3.250543e-02
##   pre_post1.pre:repl_conc2.r                         NA
##   pre_post1.pre:repl_conc3.r               1.116071e-06
##   pre_post2.post:repl_conc1.r              0.000000e+00
##   pre_post2.post:repl_conc2.r              0.000000e+00
##   pre_post2.post:repl_conc3.r              0.000000e+00
##                              difference
## pvalue                        pre_post1.pre:repl_conc3.r
##   pre_post1.pre:repl_conc1.r                  0.7628010
##   pre_post1.pre:repl_conc2.r                  0.7953064
##   pre_post1.pre:repl_conc3.r                         NA
##   pre_post2.post:repl_conc1.r                 0.0000000
##   pre_post2.post:repl_conc2.r                 0.0000000
##   pre_post2.post:repl_conc3.r                 0.0000000
##                              difference
## pvalue                        pre_post2.post:repl_conc1.r
##   pre_post1.pre:repl_conc1.r                3.291817e+00
##   pre_post1.pre:repl_conc2.r                3.324323e+00
##   pre_post1.pre:repl_conc3.r                2.529016e+00
##   pre_post2.post:repl_conc1.r                         NA
##   pre_post2.post:repl_conc2.r               1.674617e-06
##   pre_post2.post:repl_conc3.r               1.674617e-06
```

```
##                              difference
## pvalue                        pre_post2.post:repl_conc2.r
##   pre_post1.pre:repl_conc1.r                     4.054618
##   pre_post1.pre:repl_conc2.r                     4.087124
##   pre_post1.pre:repl_conc3.r                     3.291817
##   pre_post2.post:repl_conc1.r                    0.762801
##   pre_post2.post:repl_conc2.r                          NA
##   pre_post2.post:repl_conc3.r                         NaN
##                              difference
## pvalue                        pre_post2.post:repl_conc3.r
##   pre_post1.pre:repl_conc1.r                     4.054618
##   pre_post1.pre:repl_conc2.r                     4.087124
##   pre_post1.pre:repl_conc3.r                     3.291817
##   pre_post2.post:repl_conc1.r                    0.762801
##   pre_post2.post:repl_conc2.r                    0.000000
##   pre_post2.post:repl_conc3.r                          NA
##
## $PANBACT
##                              difference
## pvalue                        pre_post1.pre:repl_conc1.r
##   pre_post1.pre:repl_conc1.r                         NA
##   pre_post1.pre:repl_conc2.r                0.005944941
##   pre_post1.pre:repl_conc3.r                0.069755384
##   pre_post2.post:repl_conc1.r               0.000000000
##   pre_post2.post:repl_conc2.r               0.000000000
##   pre_post2.post:repl_conc3.r               0.000000000
##                              difference
## pvalue                        pre_post1.pre:repl_conc2.r
##   pre_post1.pre:repl_conc1.r                 -0.4426245
##   pre_post1.pre:repl_conc2.r                         NA
##   pre_post1.pre:repl_conc3.r                  0.3267128
##   pre_post2.post:repl_conc1.r                 0.0000000
##   pre_post2.post:repl_conc2.r                 0.0000000
##   pre_post2.post:repl_conc3.r                 0.0000000
##                              difference
## pvalue                        pre_post1.pre:repl_conc3.r
##   pre_post1.pre:repl_conc1.r                 -0.2863395
##   pre_post1.pre:repl_conc2.r                  0.1562850
##   pre_post1.pre:repl_conc3.r                         NA
##   pre_post2.post:repl_conc1.r                 0.0000000
##   pre_post2.post:repl_conc2.r                 0.0000000
##   pre_post2.post:repl_conc3.r                 0.0000000
##                              difference
## pvalue                        pre_post2.post:repl_conc1.r
##   pre_post1.pre:repl_conc1.r                 3.75394344
##   pre_post1.pre:repl_conc2.r                 4.19656796
##   pre_post1.pre:repl_conc3.r                 4.04028299
##   pre_post2.post:repl_conc1.r                        NA
##   pre_post2.post:repl_conc2.r                0.06975538
##   pre_post2.post:repl_conc3.r                0.06975538
##                              difference
## pvalue                        pre_post2.post:repl_conc2.r
##   pre_post1.pre:repl_conc1.r                  3.4676039
##   pre_post1.pre:repl_conc2.r                  3.9102284
```

```
##   pre_post1.pre:repl_conc3.r                        3.7539434
##   pre_post2.post:repl_conc1.r                      -0.2863395
##   pre_post2.post:repl_conc2.r                              NA
##   pre_post2.post:repl_conc3.r                             NaN
##                                difference
## pvalue                          pre_post2.post:repl_conc3.r
##   pre_post1.pre:repl_conc1.r                        3.4676039
##   pre_post1.pre:repl_conc2.r                        3.9102284
##   pre_post1.pre:repl_conc3.r                        3.7539434
##   pre_post2.post:repl_conc1.r                      -0.2863395
##   pre_post2.post:repl_conc2.r                       0.0000000
##   pre_post2.post:repl_conc3.r                              NA
```

Experiment 2 (Figure 1 - B)

Test what happens when we increase the number of bacterial contaminants in Milli-Q water. Two DNA extraction methods tested (type variable) Species involved = Legionella pneumophila, Chlostridium perfringens, Lactobacillus rhamnosus, Lactobacillus plantarum, Escherichia coli, Pseudomonas aeruginosa, Bifidobacterium longum, Bifidobacterium lactis, Lactobacillus reuteri, Salmonella choleraesuis, Staphilococcus aerus, Enterococcus hirae and panbacterial marker

```
exp2.d <- read.csv("exp_2.csv")
```

```
exp2.m= mcmc.qpcr(
    fixed="pre_post+type+pre_post:type",
    burnin=4000,
    data= exp2.d)
```

```
summary(exp2.m)
```

```
##
##  Iterations = 4001:12991
##  Thinning interval  = 10
##  Sample size  = 900
##
##  DIC: 2164.834
##
##  G-structure:  ~sample
##
##        post.mean  l-95% CI u-95% CI eff.samp
## sample 5.501e-43 5.503e-82 1.83e-47        0
##
##  R-structure:  ~idh(gene):units
##
##                   post.mean l-95% CI u-95% CI eff.samp
## geneBLACT.units      0.6248   0.2892   1.0424    720.6
## geneBLONG.units      0.6461   0.2984   1.1050   1185.9
## geneECOLI.units      0.6414   0.3271   1.1375    900.0
## geneENTERO.units     0.6531   0.2929   1.1305    900.0
## geneLEGIO.units      0.6381   0.3268   1.1053    900.0
## geneLPLANT.units     0.6439   0.3068   1.0448    975.2
```

```
## geneLREU.units       0.6158   0.2812   1.0224    900.0
## geneLRHAM.units      0.6472   0.3149   1.1244    900.0
## genePANBACT.units    0.5775   0.2939   0.9669    900.0
## genePSEUDO.units     0.6019   0.2987   0.9642    900.0
## geneSALMO.units      0.6442   0.2733   1.0723    900.0
## geneSTAPH.units      0.5767   0.2807   0.9552    900.0
##
##  Location effects: count ~ 0 + gene + +gene:pre_post + gene:type + gene:pre_post:type
##
##                                  post.mean l-95% CI u-95% CI eff.samp
## geneBLACT                          5.53441  4.55947  6.37075    900.0
## geneBLONG                          1.15504  0.02808  2.20111    757.2
## geneECOLI                          8.24818  7.40880  9.13268    811.8
## geneENTERO                         9.03803  8.12216  9.95701    900.0
## geneLEGIO                          7.83918  6.91064  8.71204    962.7
## geneLPLANT                         6.04435  5.02633  6.92075   1093.6
## geneLREU                           3.19152  2.26978  4.11653   1003.8
## geneLRHAM                          8.37412  7.37784  9.22587    795.4
## genePANBACT                       11.42836 10.60123 12.35123    900.0
## genePSEUDO                        11.59556 10.75099 12.46935    900.0
## geneSALMO                          9.58729  8.82548 10.55483    900.0
## geneSTAPH                          8.84236  7.96108  9.70786    731.6
## geneBLACT:pre_post2.post           1.84443  0.61788  3.04010    900.0
## geneBLONG:pre_post2.post           1.88392  0.44698  3.26367    900.0
## geneECOLI:pre_post2.post           2.24881  1.14239  3.40377    808.1
## geneENTERO:pre_post2.post          1.97226  0.75802  3.15504   1368.6
## geneLEGIO:pre_post2.post           2.28874  0.76323  3.38343    784.2
## geneLPLANT:pre_post2.post          2.17764  1.02095  3.32997    772.2
## geneLREU:pre_post2.post            2.19048  1.13580  3.59700    900.0
## geneLRHAM:pre_post2.post           2.90610  1.49066  3.95926    900.0
## genePANBACT:pre_post2.post         1.84191  0.85287  3.21396    900.0
## genePSEUDO:pre_post2.post          2.43284  1.26590  3.58204    900.0
## geneSALMO:pre_post2.post           2.47576  1.30077  3.59388    900.0
## geneSTAPH:pre_post2.post           2.61050  1.50795  3.83812    900.0
## geneBLACT:typeB                    1.41422  0.07735  2.65730    900.0
## geneBLONG:typeB                    1.36482  0.06661  2.78739   1005.0
## geneECOLI:typeB                    3.22149  1.83378  4.36595    787.2
## geneENTERO:typeB                   0.25076 -1.10214  1.75170    900.0
## geneLEGIO:typeB                    3.23155  2.04249  4.64399    900.0
## geneLPLANT:typeB                   1.62940  0.27362  2.75552    808.0
## geneLREU:typeB                     3.41768  2.10009  4.65534    900.0
## geneLRHAM:typeB                    1.00940 -0.28312  2.12501   1030.8
## genePANBACT:typeB                  0.19051 -0.87211  1.34540    900.0
## genePSEUDO:typeB                   1.85133  0.49011  2.93953    900.0
## geneSALMO:typeB                    1.01183 -0.31120  2.20432    866.9
## geneSTAPH:typeB                    3.07389  2.02941  4.28095    824.2
## geneBLACT:pre_post2.post:typeB     0.28873 -1.22486  2.20077    900.0
## geneBLONG:pre_post2.post:typeB     0.18551 -1.77151  1.78958    900.0
## geneECOLI:pre_post2.post:typeB     0.29384 -1.27505  2.31674    900.0
## geneENTERO:pre_post2.post:typeB   -0.20787 -1.93785  1.78830    800.5
## geneLEGIO:pre_post2.post:typeB    -0.13294 -1.91940  1.73182    738.9
## geneLPLANT:pre_post2.post:typeB   -0.74444 -2.20931  1.09852    900.0
## geneLREU:pre_post2.post:typeB      0.02094 -1.61982  1.74912   1029.8
## geneLRHAM:pre_post2.post:typeB    -0.22100 -1.82639  1.50143    900.0
```

```
## genePANBACT:pre_post2.post:typeB   0.26993 -1.28015  1.88436    900.0
## genePSEUDO:pre_post2.post:typeB   -0.08034 -1.72598  1.52064    900.0
## geneSALMO:pre_post2.post:typeB    -0.12174 -2.24908  1.53849   1021.1
## geneSTAPH:pre_post2.post:typeB    -0.38722 -1.96323  1.24722    900.0
##                                    pMCMC
## geneBLACT                         < 0.001 **
## geneBLONG                         0.04444 *
## geneECOLI                         < 0.001 **
## geneENTERO                        < 0.001 **
## geneLEGIO                         < 0.001 **
## geneLPLANT                        < 0.001 **
## geneLREU                          < 0.001 **
## geneLRHAM                         < 0.001 **
## genePANBACT                       < 0.001 **
## genePSEUDO                        < 0.001 **
## geneSALMO                         < 0.001 **
## geneSTAPH                         < 0.001 **
## geneBLACT:pre_post2.post          0.00667 **
## geneBLONG:pre_post2.post          0.01556 *
## geneECOLI:pre_post2.post          < 0.001 **
## geneENTERO:pre_post2.post         < 0.001 **
## geneLEGIO:pre_post2.post          0.00222 **
## geneLPLANT:pre_post2.post         < 0.001 **
## geneLREU:pre_post2.post           0.00222 **
## geneLRHAM:pre_post2.post          < 0.001 **
## genePANBACT:pre_post2.post        0.00667 **
## genePSEUDO:pre_post2.post         < 0.001 **
## geneSALMO:pre_post2.post          < 0.001 **
## geneSTAPH:pre_post2.post          < 0.001 **
## geneBLACT:typeB                   0.05111 .
## geneBLONG:typeB                   0.05333 .
## geneECOLI:typeB                   < 0.001 **
## geneENTERO:typeB                  0.73333
## geneLEGIO:typeB                   < 0.001 **
## geneLPLANT:typeB                  0.00889 **
## geneLREU:typeB                    < 0.001 **
## geneLRHAM:typeB                   0.10889
## genePANBACT:typeB                 0.72889
## genePSEUDO:typeB                  0.01111 *
## geneSALMO:typeB                   0.13778
## geneSTAPH:typeB                   < 0.001 **
## geneBLACT:pre_post2.post:typeB    0.76444
## geneBLONG:pre_post2.post:typeB    0.85778
## geneECOLI:pre_post2.post:typeB    0.73778
## geneENTERO:pre_post2.post:typeB   0.81556
## geneLEGIO:pre_post2.post:typeB    0.87333
## geneLPLANT:pre_post2.post:typeB   0.40222
## geneLREU:pre_post2.post:typeB     1.00000
## geneLRHAM:pre_post2.post:typeB    0.81778
## genePANBACT:pre_post2.post:typeB  0.72222
## genePSEUDO:pre_post2.post:typeB   0.89778
## geneSALMO:pre_post2.post:typeB    0.90889
## geneSTAPH:pre_post2.post:typeB    0.60889
## ---
```

```
## Signif. codes:  0 '***' 0.001 '**' 0.01 '*' 0.05 '.' 0.1 ' ' 1
```

```
s2$summary
```

```
##       gene pre_post type      mean        sd      lower     upper
## 1    BLACT    1.pre    A  7.984464 0.6646611  6.9473830  9.091158
## 2    BLACT    1.pre    B 10.024757 0.6621872  8.9045033 11.110241
## 3    BLACT   2.post    A 10.645418 0.5896825  9.6737537 11.612667
## 4    BLACT   2.post    B 13.102261 0.6740097 12.0356617 14.169055
## 5    BLONG    1.pre    A  1.666372 0.8149226  0.3430308  2.948005
## 6    BLONG    1.pre    B  3.635385 0.6151337  2.6423582  4.664987
## 7    BLONG   2.post    A  4.384296 0.6322056  3.3313405  5.399292
## 8    BLONG   2.post    B  6.620940 0.6614099  5.5787105  7.714753
## 9    ECOLI    1.pre    A 11.899614 0.6374333 10.8816237 12.929985
## 10   ECOLI    1.pre    B 16.547245 0.6748082 15.4111408 17.695933
## 11   ECOLI   2.post    A 15.143956 0.5765010 14.2059287 16.106102
## 12   ECOLI   2.post    B 20.215506 0.6530669 19.1584105 21.255979
## 13  ENTERO    1.pre    A 13.039118 0.6504445 11.9686782 14.084416
## 14  ENTERO    1.pre    B 13.400892 0.8119219 12.0832977 14.701124
## 15  ENTERO   2.post    A 15.884481 0.5819299 14.9244051 16.846025
## 16  ENTERO   2.post    B 15.946360 0.6879495 14.7387138 17.007155
## 17   LEGIO    1.pre    A 11.309540 0.6762446 10.2006831 12.399585
## 18   LEGIO    1.pre    B 15.971685 0.6512384 14.9252835 17.034532
## 19   LEGIO   2.post    A 14.611492 0.6592717 13.5205241 15.670623
## 20   LEGIO   2.post    B 19.081844 0.6856395 17.9897947 20.212555
## 21  LPLANT    1.pre    A  8.720154 0.6816605  7.5912268  9.846501
## 22  LPLANT    1.pre    B 11.070884 0.5529007 10.1834106 11.972677
## 23  LPLANT   2.post    A 11.861820 0.5908180 10.9007943 12.844101
## 24  LPLANT   2.post    B 13.138549 0.6446521 12.0477950 14.186119
## 25    LREU    1.pre    A  4.604387 0.6950083  3.4413271  5.764895
## 26    LREU    1.pre    B  9.535060 0.6540347  8.3860856 10.552101
## 27    LREU   2.post    A  7.764588 0.5783743  6.8526749  8.747581
## 28    LREU   2.post    B 12.725475 0.6461625 11.6475477 13.771272
## 29   LRHAM    1.pre    A 12.081296 0.6747803 10.9861602 13.215670
## 30   LRHAM    1.pre    B 13.537553 0.5854783 12.5526141 14.482647
## 31   LRHAM   2.post    A 16.273908 0.5925214 15.2964808 17.252778
## 32   LRHAM   2.post    B 17.411330 0.5945858 16.4277533 18.407432
## 33 PANBACT    1.pre    A 16.487634 0.6326796 15.4551313 17.511727
## 34 PANBACT    1.pre    B 16.762476 0.5315535 15.9075093 17.668141
## 35 PANBACT   2.post    A 19.144946 0.5624180 18.2299961 20.081512
## 36 PANBACT   2.post    B 19.809217 0.5693890 18.8787944 20.767641
## 37  PSEUDO    1.pre    A 16.728863 0.6331559 15.6453385 17.773060
## 38  PSEUDO    1.pre    B 19.399774 0.6279812 18.3150381 20.417871
## 39  PSEUDO   2.post    A 20.238712 0.5406748 19.4027582 21.113967
## 40  PSEUDO   2.post    B 22.793719 0.6310858 21.7648488 23.856399
## 41   SALMO    1.pre    A 13.831540 0.6429525 12.8351411 14.866926
## 42   SALMO    1.pre    B 15.291309 0.6977995 14.1434979 16.402495
## 43   SALMO   2.post    A 17.403311 0.5667682 16.5372043 18.354044
## 44   SALMO   2.post    B 18.687449 0.8525147 17.2607404 20.014309
## 45   STAPH    1.pre    A 12.756835 0.6436652 11.6878426 13.824361
## 46   STAPH    1.pre    B 17.191527 0.5374026 16.3649800 18.092354
## 47   STAPH   2.post    A 16.522985 0.5387330 15.6161628 17.370977
## 48   STAPH   2.post    B 20.399034 0.5459288 19.5187193 21.306214
```

```
s2$geneWise
```

```
## $BLACT
##                       difference
## pvalue                 pre_post1.pre:typeA pre_post1.pre:typeB
##   pre_post1.pre:typeA                   NA          2.04029344
##   pre_post1.pre:typeB         3.146657e-02                  NA
##   pre_post2.post:typeA        2.279645e-03          0.49074343
##   pre_post2.post:typeB        3.841934e-08          0.00101611
##                       difference
## pvalue                 pre_post2.post:typeA pre_post2.post:typeB
##   pre_post1.pre:typeA           2.660954688             5.117797
##   pre_post1.pre:typeB           0.620661245             3.077503
##   pre_post2.post:typeA                   NA             2.456842
##   pre_post2.post:typeB          0.005430469                   NA
##
## $BLONG
##                       difference
## pvalue                 pre_post1.pre:typeA pre_post1.pre:typeB
##   pre_post1.pre:typeA                   NA         1.969012992
##   pre_post1.pre:typeB         5.008546e-02                  NA
##   pre_post2.post:typeA        8.452932e-03         0.413607339
##   pre_post2.post:typeB        2.533786e-06         0.001038322
##                       difference
## pvalue                 pre_post2.post:typeA pre_post2.post:typeB
##   pre_post1.pre:typeA             2.7179245             4.954569
##   pre_post1.pre:typeB             0.7489115             2.985556
##   pre_post2.post:typeA                   NA             2.236644
##   pre_post2.post:typeB            0.0147003                   NA
##
## $ECOLI
##                       difference
## pvalue                 pre_post1.pre:typeA pre_post1.pre:typeB
##   pre_post1.pre:typeA                   NA         4.647630970
##   pre_post1.pre:typeB         6.449701e-07                  NA
##   pre_post2.post:typeA        1.573773e-04         0.128562082
##   pre_post2.post:typeB        0.000000e+00         0.000122102
##                       difference
## pvalue                 pre_post2.post:typeA pre_post2.post:typeB
##   pre_post1.pre:typeA          3.244342e+00             8.315892
##   pre_post1.pre:typeB         -1.403289e+00             3.668261
##   pre_post2.post:typeA                   NA             5.071550
##   pre_post2.post:typeB         8.858082e-09                   NA
##
## $ENTERO
##                       difference
## pvalue                 pre_post1.pre:typeA pre_post1.pre:typeB
##   pre_post1.pre:typeA                   NA          0.36177372
##   pre_post1.pre:typeB          0.730761239                  NA
##   pre_post2.post:typeA         0.001204542          0.01388823
##   pre_post2.post:typeB         0.002375783          0.01540348
##                       difference
## pvalue                 pre_post2.post:typeA pre_post2.post:typeB
```

```
##   pre_post1.pre:typeA            2.8453627          2.90724197
##   pre_post1.pre:typeB            2.4835890          2.54546825
##   pre_post2.post:typeA                  NA          0.06187925
##   pre_post2.post:typeB           0.9454812                  NA
##
## $LEGIO
##                       difference
## pvalue                 pre_post1.pre:typeA pre_post1.pre:typeB
##   pre_post1.pre:typeA                   NA         4.662145404
##   pre_post1.pre:typeB         9.120503e-07                  NA
##   pre_post2.post:typeA        5.694962e-04         0.156501242
##   pre_post2.post:typeB        4.440892e-16         0.001074261
##                       difference
## pvalue                 pre_post2.post:typeA pre_post2.post:typeB
##   pre_post1.pre:typeA          3.301952e+00             7.772304
##   pre_post1.pre:typeB         -1.360193e+00             3.110159
##   pre_post2.post:typeA                   NA             4.470352
##   pre_post2.post:typeB         4.099701e-06                   NA
##
## $LPLANT
##                       difference
## pvalue                 pre_post1.pre:typeA pre_post1.pre:typeB
##   pre_post1.pre:typeA                   NA          2.35072947
##   pre_post1.pre:typeB         9.932671e-03                  NA
##   pre_post2.post:typeA        3.609913e-04          0.32135314
##   pre_post2.post:typeB        2.337382e-06          0.01540598
##                       difference
## pvalue                 pre_post2.post:typeA pre_post2.post:typeB
##   pre_post1.pre:typeA             3.1416656             4.418395
##   pre_post1.pre:typeB             0.7909361             2.067666
##   pre_post2.post:typeA                   NA             1.276730
##   pre_post2.post:typeB            0.1437629                   NA
##
## $LREU
##                       difference
## pvalue                 pre_post1.pre:typeA pre_post1.pre:typeB
##   pre_post1.pre:typeA                   NA        4.9306734370
##   pre_post1.pre:typeB         2.438117e-07                  NA
##   pre_post2.post:typeA        4.656414e-04        0.0436719811
##   pre_post2.post:typeB        0.000000e+00        0.0003630308
##                       difference
## pvalue                 pre_post2.post:typeA pre_post2.post:typeB
##   pre_post1.pre:typeA          3.160202e+00             8.121088
##   pre_post1.pre:typeB         -1.770472e+00             3.190414
##   pre_post2.post:typeA                   NA             4.960886
##   pre_post2.post:typeB         2.100235e-08                   NA
##
## $LRHAM
##                       difference
## pvalue                 pre_post1.pre:typeA pre_post1.pre:typeB
##   pre_post1.pre:typeA                   NA        1.456256e+00
##   pre_post1.pre:typeB          1.06436e-01                  NA
##   pre_post2.post:typeA         2.38325e-06        6.167269e-04
##   pre_post2.post:typeB         6.61621e-09        4.404743e-06
```

```
##                         difference
## pvalue                   pre_post2.post:typeA pre_post2.post:typeB
##   pre_post1.pre:typeA               4.1926111             5.330034
##   pre_post1.pre:typeB               2.7363548             3.873778
##   pre_post2.post:typeA                     NA             1.137423
##   pre_post2.post:typeB              0.1750064                   NA
##
## $PANBACT
##                         difference
## pvalue                   pre_post1.pre:typeA pre_post1.pre:typeB
##   pre_post1.pre:typeA                     NA        2.748421e-01
##   pre_post1.pre:typeB         7.386967e-01                  NA
##   pre_post2.post:typeA        1.862042e-03        1.631460e-03
##   pre_post2.post:typeB        6.961962e-05        5.638729e-05
##                         difference
## pvalue                   pre_post2.post:typeA pre_post2.post:typeB
##   pre_post1.pre:typeA               2.6573125            3.3215828
##   pre_post1.pre:typeB               2.3824703            3.0467407
##   pre_post2.post:typeA                     NA            0.6642703
##   pre_post2.post:typeB              0.4069676                   NA
##
## $PSEUDO
##                         difference
## pvalue                   pre_post1.pre:typeA pre_post1.pre:typeB
##   pre_post1.pre:typeA                     NA        2.6709108665
##   pre_post1.pre:typeB         3.670747e-03                  NA
##   pre_post2.post:typeA        2.199708e-05        0.3072013259
##   pre_post2.post:typeB        1.577027e-11        0.0001524743
##                         difference
## pvalue                   pre_post2.post:typeA pre_post2.post:typeB
##   pre_post1.pre:typeA             3.509848720             6.064856
##   pre_post1.pre:typeB             0.838937854             3.393945
##   pre_post2.post:typeA                     NA             2.555007
##   pre_post2.post:typeB            0.001762882                   NA
##
## $SALMO
##                         difference
## pvalue                   pre_post1.pre:typeA pre_post1.pre:typeB
##   pre_post1.pre:typeA                     NA         1.459769135
##   pre_post1.pre:typeB         1.285778e-01                  NA
##   pre_post2.post:typeA        3.147015e-05         0.022448328
##   pre_post2.post:typeB        5.508009e-06         0.001256584
##                         difference
## pvalue                   pre_post2.post:typeA pre_post2.post:typeB
##   pre_post1.pre:typeA                3.571772             4.855909
##   pre_post1.pre:typeB                2.112003             3.396140
##   pre_post2.post:typeA                     NA             1.284137
##   pre_post2.post:typeB               0.225616                   NA
##
## $STAPH
##                         difference
## pvalue                   pre_post1.pre:typeA pre_post1.pre:typeB
##   pre_post1.pre:typeA                     NA        4.434692e+00
##   pre_post1.pre:typeB         1.358361e-07                  NA
```

```
##   pre_post2.post:typeA        1.114831e-05        3.661639e-01
##   pre_post2.post:typeB        0.000000e+00        2.013383e-05
##                         difference
## pvalue                   pre_post2.post:typeA pre_post2.post:typeB
##   pre_post1.pre:typeA           3.766150e+00             7.642198
##   pre_post1.pre:typeB          -6.685419e-01             3.207507
##   pre_post2.post:typeA                    NA             3.876049
##   pre_post2.post:typeB          6.997796e-07                   NA
```

Experiment 3 (Fig 1 - C)

Test what happens when we use the drinking water with bacterial contaminants (Same of Exp.1) Species involved = Legionella pneumophila, Chlostridium perfringens, Lactobacillus rhamnosus, Lactobacillus plantarum and panbacterial marker)

```
exp3.d <- read.csv("exp_3.csv")
```

```
exp3.m= mcmc.qpcr(
    fixed="pre_post",
    burnin=3000,
    data= exp3.d)
```

```
s3$summary
```

```
##       gene pre_post      mean        sd     lower     upper
## 1  CLOSTRI    1.pre 10.808474 0.2411359 10.407797 11.214501
## 2  CLOSTRI   2.post 15.234861 0.2374629 14.871056 15.625878
## 3    LEGIO    1.pre 15.497875 0.2291278 15.124388 15.851821
## 4    LEGIO   2.post 19.777084 0.2307583 19.408584 20.165622
## 5   LPLANT    1.pre  6.028480 0.2381250  5.642279  6.413935
## 6   LPLANT   2.post 10.664575 0.2289002 10.294858 11.039848
## 7    LRHAM    1.pre  7.319097 0.2389649  6.935353  7.692801
## 8    LRHAM   2.post 11.357681 0.2359161 10.963814 11.752516
## 9  PANBACT    1.pre 12.760869 0.2249581 12.405493 13.121071
## 10 PANBACT   2.post 16.706885 0.2416055 16.326909 17.102023
```

```
summary(exp3.m)
```

```
##
##  Iterations = 3001:12991
##  Thinning interval  = 10
##  Sample size  = 1000
##
##  DIC: 1129.516
##
##  G-structure:  ~sample
##
##        post.mean  l-95% CI  u-95% CI eff.samp
## sample 3.602e-15 3.824e-60 4.577e-17        0
##
```

```
##  R-structure:  ~idh(gene):units
##
##                   post.mean l-95% CI u-95% CI eff.samp
## geneCLOSTRI.units    0.2490  0.11440   0.4352     1000
## geneLEGIO.units      0.2320  0.11316   0.3978     1000
## geneLPLANT.units     0.2384  0.10585   0.3983     1012
## geneLRHAM.units      0.2326  0.09759   0.3984     1000
## genePANBACT.units    0.2352  0.10850   0.4053     1000
##
##  Location effects: count ~ 0 + gene + +gene:pre_post
##
##                            post.mean l-95% CI u-95% CI eff.samp  pMCMC
## geneCLOSTRI                    7.492    7.155    7.813     1335 <0.001 ***
## geneLEGIO                     10.742   10.397   11.018     1000 <0.001 ***
## geneLPLANT                     4.179    3.833    4.479     1000 <0.001 ***
## geneLRHAM                      5.073    4.785    5.430     1000 <0.001 ***
## genePANBACT                    8.845    8.532    9.125     1000 <0.001 ***
## geneCLOSTRI:pre_post2.post     3.068    2.600    3.531     1000 <0.001 ***
## geneLEGIO:pre_post2.post       2.966    2.551    3.469     1000 <0.001 ***
## geneLPLANT:pre_post2.post      3.213    2.746    3.682     1000 <0.001 ***
## geneLRHAM:pre_post2.post       2.799    2.345    3.285     1000 <0.001 ***
## genePANBACT:pre_post2.post     2.735    2.293    3.193     1000 <0.001 ***
## ---
## Signif. codes:  0 '***' 0.001 '**' 0.01 '*' 0.05 '.' 0.1 ' ' 1
```

```
s3$geneWise
```

```
## $CLOSTRI
##         difference
## pvalue   1.pre   2.post
##   1.pre     NA 4.426387
##   2.post     0       NA
##
## $LEGIO
##         difference
## pvalue   1.pre   2.post
##   1.pre     NA 4.279209
##   2.post     0       NA
##
## $LPLANT
##         difference
## pvalue   1.pre   2.post
##   1.pre     NA 4.636095
##   2.post     0       NA
##
## $LRHAM
##         difference
## pvalue   1.pre   2.post
##   1.pre     NA 4.038584
##   2.post     0       NA
##
## $PANBACT
##         difference
## pvalue   1.pre   2.post
```

```
##   1.pre     NA 3.946016
##   2.post     0       NA
```

Experiment 4 (Fig. 1 - D)

Test what happens when we use the drinking water without bacterial contaminants Species involved = panbacterial marker

```
exp4.d<- read.csv("exp_4.csv")
exp.4.post = subset(exp4.d, pre_post=="2.post")
```

```
exp.4.m = glm(count ~ type + exp +type:exp, data= exp.4.post, family=poisson())
```

```
summary(exp.4.m)
```

```
##
## Call:
## glm(formula = count ~ type + exp + type:exp, family = poisson(),
##     data = exp.4.post)
##
## Deviance Residuals:
##     Min       1Q   Median       3Q      Max
## -6.0691  -0.4200   0.0000   0.3125   5.9805
##
## Coefficients:
##               Estimate Std. Error z value Pr(>|z|)
## (Intercept)     1.4663     0.2774   5.287 1.24e-07 ***
## typeB           5.9876     0.2777  21.562  < 2e-16 ***
## exp4.env        0.3254     0.3640   0.894    0.371
## exp9.env        0.6131     0.3444   1.780    0.075 .
## typeB:exp4.env  -0.4591     0.3645  -1.259    0.208
## typeB:exp9.env  -1.5994     0.3454  -4.630 3.65e-06 ***
## ---
## Signif. codes:  0 '***' 0.001 '**' 0.01 '*' 0.05 '.' 0.1 ' ' 1
##
## (Dispersion parameter for poisson family taken to be 1)
##
##     Null deviance: 17316.939  on 17  degrees of freedom
## Residual deviance:    95.351  on 12  degrees of freedom
## AIC: 220.22
##
## Number of Fisher Scoring iterations: 4
```
