# Supplementary material for: One step forwards for the routine use of high‐throughput DNA sequencing in environmental monitoring. An efficient and standardizable method to maximize the detection of environmental bacteria
Source: Microbiologyopen. 2016 Oct 27;6(1):e00421. doi: 10.1002/mbo3.421 (PMC5300880; doi:10.1002/mbo3.421)
Supplement: Supplementary file 2 [file MBO3-6-0-s002.docx]

**Supplementary Tables**

**Table S01**

| **Bacteria** | **GRAM staining** | **OD_600_** | **CFU (10^5 mL^-1^)** |
| --- | --- | --- | --- |
| *Escherichia coli* | GRAM - | 1.8 | 367 |
| *Salmonella choleraesuis* | GRAM - | 2.4 | 506 |
| *Legionella pneumophila* | GRAM - | 0.13 | 315 |
| *Pseudomonas aeruginosa* | GRAM - | 2.8 | 427 |
| *Clostridium perfringens* | GRAM + | NA | NA |
| *Staphylococcus aureus* | GRAM + | 2.3 | 784 |
| *Enterococcus hirae* | GRAM + | NA | NA |
| *Lactobacillus rhamnosus* | GRAM + | 3.1 | NA |
| *Lactobacillus plantarum* | GRAM + | 2.8 | NA |
| *Lactobacillus reuteri* | GRAM + | 3.1 | NA |
| *Bifidobacterium lactis* | GRAM + | 0.08 | NA |
| *Bifidobacterium longum* | GRAM + | 2.7 | NA |

Table S01. Optical densities (OD_600_) and/or CFU of each monoculture. One mL of monoculture is used to create the mock communities listed in table S02. Only optical densities were reported for damage cultures. NA is for Not Available data.

**Table S02**

| **Experiment #1 mix** | *Legionella pneumophila*, *Clostridium perfringens***,** *Lactobacillus rhamnosus*, *Lactobacillus plantarum***.** |
| --- | --- |
| **Experiment #2 mix** | *Escherichia coli*, *Salmonella choleraesuis*, *Legionella pneumophila*, *Pseudomonas aeruginosa*, *Clostridium perfringens*, *Staphylococcus aureus*, *Enterococcus hirae*, *Lactobacillus rhamnosus*, *Lactobacillus plantarum*, *Lactobacillus reuteri*, *Bifidobacterium lactis*, *Bifidobacterium longum*. |
| **Experiment #3 mix** | *Legionella pneumophila*, *Clostridium perfringens***,** *Lactobacillus rhamnosus*, *Lactobacillus plantarum***.** |

Table S02: Bacterial composition of mock communities used for the artificial contamination of water samples.

**Table S03**

| **Sample name** | **T (°C)** | **pH** | **Conductivity (µS/cm)** | **Cl_2_ (mg/L)** |
| --- | --- | --- | --- | --- |
| **D-00** | 13 | 7.5 | 700 | 0.03 |
| **D-01** | 12.9 | 7.45 | 704 | 0.02 |
| **D-02** | 12.9 | 7.65 | 617 | 0.03 |
| **D-03** | 16.3 | 7.53 | 700 | 0.04 |

Table S03: Characteristics of environmental samples (drinking water) used. D-00: drinking water sample used in Exp - 3. D-01, D-02, D-03: drinking water samples used in the Exp - 4.

**Table S04**

| **Target** | **primers** | **sequence** | **gene** | **T annealing** | **Reference** |
| --- | --- | --- | --- | --- | --- |
| *E. coli* | ColiF | CATGCCGCGTGTATGAAGAA | 16S | 60 °C | Huijsdens et al., 2002 |
|  | ColiR | CGGGTAACGTCAATGAGCAAA |  |  |  |
| *S. choleraesuis* | SAL1410f | GGTCTGCTGTACTCCACCTTCAG | bipA | 60 °C | Calvò et al., 2008 |
|  | SAL1494r | TTGGAGATCAGTACGCCGTTCT |  |  |  |
| *L. pneumophila* | JFP | AGGGTTGATAGGTTAAGAGC | 16S-23S | 60 °C | Devos et al., 2005 |
|  | JRP | CCAACAGCTAGTTGACATCG |  |  |  |
| *P. aeruginosa* | P.aer_F | AATTCGGCAAATTTGCTGCG | oprL | 60 °C | Wong et al., 2014 |
|  | P.aer_R | GGAGCTGTCGTACTCGAAGT |  |  |  |
| *C. perfringens* | ClperF | GCATGAGTCATAGTTGGGATGATT | plc | 60 °C | Shannon et al., 2007 |
|  | ClperR | CCTGCTGTTCCTTTTTGAGAGTTAG |  |  |  |
| *S. aureus* | S.aur_F | GCGATTGATGGTGATACGGTT | nucA | 60 °C | Brakstad et al., 1992 |
|  | S.aur_R | AGCCAAGCCTTGACGAACTAAAGC |  |  |  |
| *E. hirae* | EC_F | AGAAATTCCAAACGAACTTG | 23S | 55 °C | He et al., 2005 |
|  | EC_R | CAGTGCTCTACCTCCATCATT |  |  |  |
| *L. rhamnosus* | LrhamF | TGCTTGCATCTTGATTTAATTTTG | 16S | 60 °C | Zhang et al., 2012 |
|  | LrhamR | GGTTCTTGGATYTATGCGGTATTAG |  |  |  |
| *L. plantarum* | LplF | CCAGTTGGTTCCATAAGTTG | 16S-23S | 60 °C | Our lab |
|  | LplR | GTTTCAATGACGACTAACGTC |  |  |  |
| *L. reuteri* | LreuF | GGAACCTACACATCGAAG | 16S | 60 °C | Our lab |
|  | LreuR | CAAATAACGCGGTGTTCTC |  |  |  |
| *B. lactis* | BlacF | GCACGGTTTTGTGGCTGG | 16S | 60 °C | Our lab |
|  | BlacR | GACCTGGGGGACACACTG |  |  |  |
| *B. longum* | BloF | CAGTTGATCGCATGGTCTT | 16S | 60 °C | Malinen et al.,  2005 |
|  | BloR | TACCCGTCGAAGCCAC |  |  |  |
| panbacterial | 341F | CCTACGGGNGGCWGCAG | 16S | 55 °C | 16S Metagenomic Sequencing Library Preparation (Illumina sequencing protocol); Herlemann et al., 2011 |
|  | 805R | GACTACHVGGGTATCTAATCC |  |  |  |

Table S04. Primers used for the quantification of target bacteria through qPCR. For each target primer sequences, gene, annealing temperatures and references are reported.

**Table S05**

| **Target** | **Recovery efficiency A (%)** | **s.d.** | **Recovery efficiency B (%)** | **s.d.** |
| --- | --- | --- | --- | --- |
| *E. coli* | 95 | 0.02 | 131 | 0.91 |
| *S. choleraesuis* | 117 | 0.04 | 100 | 0.83 |
| *L. pneumophila* | 97 | 0.13 | 88 | 0.01 |
| *P. aeruginosa* | 113 | 0.01 | 106 | 0.1 |
| *C. perfringens* | NA | NA | 49 | 2.34 |
| *S. aureus* | 135 | 0.14 | 94 | 0.38 |
| *E. hirae* | 71 | 0.01 | 63 | 0.05 |
| *L. rhamnosus* | 180 | 0.17 | 151 | 1.2 |
| *L. plantarum* | 88 | 0.09 | 41 | 1.39 |
| *L. reuteri* | 89 | 0.11 | 93 | 0.01 |
| *B. lactis* | 61 | 0.04 | 80 | 0.11 |
| *B. longum* | 62 | 0.44 | 79 | 0.33 |
| panbacterial | 65 | 0.04 | 83 | 0.3 |

Table S05. Recovery efficiencies for Exp - 2. A: One-step DNA extraction. B: Automated DNA extraction. s.d.: standard deviation

**Table S06**

| Target | **Recovery efficiency (%)** | **s. d.** |
| --- | --- | --- |
| *L. pneumophila* | 98 | 1.57 |
| *C. perfringens* | 107 | 1.64 |
| *L. rhamnosus* | 82 | 1.55 |
| *L. plantarum* | 123 | 9.65 |
| *panbacterial* | 77 | 1.56 |

Table S06. Recovery efficiencies for artificially contaminated drinking water (Exp - 3). s. d.: standard deviation.

**Table S07**

| **Sample name** | **DNA extraction** | **target** | **log_2_(counts)**  **pre** | **log_2_(counts)**  **post** | **sd** |
| --- | --- | --- | --- | --- | --- |
| D-01 B | automated | panbacteria | NA | 14.19 | 0.73 |
| D-02 B | automated | panbacteria | NA | 11.02 | 4.35 |
| D-03 B | automated | panbacteria | NA | 8.11 | 3.87 |
| D-01 A | One-step lysis | panbacteria | NA | 5.8 | 0.62 |
| D-02 A | One-step lysis | panbacteria | NA | 6.09 | 0.45 |
| D-03 A | One-step lysis | panbacteria | NA | 6.37 | 0.01 |

Table S07. Counts, expressed as log_2_ values, for drinking water samples, pre and post concentration, comparing two different DNA extraction methods.

**Table S08**

| **Sample name** | **Library quantification**  **(pg/µL DNA)** | **observed OTUs (unique clusters - 100%)** | **Observed OTUs (unique clusters - 100%) > 0.01 %** | **Shared OTUs > 0.01 %** | **reads** |
| --- | --- | --- | --- | --- | --- |
| D-01B | 3530 | 3140 | 117 | 116 | 43121 |
| D-01B |  | 3688 | 123 |  | 63904 |
| D-02 B | 71.4 | 872 | 111 | 111 | 4478 |
| D-02 B |  | 1083 | 114 |  | 7199 |
| D-03 B | 2350 | 1858 | 111 | 111 | 9036 |
| D-03 B |  | 2350 | 111 |  | 14958 |

Table S08. Library quantification and sequencing results obtained from drinking water samples.

**Table S09**

| **Bacteria** | **% Live bacteria**  **pre** | **% Live bacteria**  **post** | **Bacteria**  **in filtrate** |
| --- | --- | --- | --- |
| *E. coli* | 100 | NA | NA |
| *S. choleraesuis* | 100 | NA | NA |
| *L. pneumophila* | 100 | NA | NA |
| *P. aeruginosa* | 100 | NA | NA |
| *C. perfringens* | 100 | NA | NA |
| *S. aureus* | 100 | NA | NA |
| *E. hirae* | 100 | NA | NA |
| *L. rhamnosus* | 0 | NA | NA |
| *L. plantarum* | 0 | NA | NA |
| *L. reuteri* | 0 | NA | NA |
| *B. lactis* | 0 | NA | NA |
| *B. longum* | 0 | NA | NA |
| Mix Exp - 1 | 70 ± 5 | 70 ± 6 | 0 |
| Mix Exp - 2 | 60± 7 | 60 ± 5 | 0 |
| Mix Exp - 3 | 70 ± 5 | 70± 8 | 0 |
| Exp - 4 | 90 ± 9 | 90± 6 | 0 |

Table S09. Live/dead ratios of starting bacterial monocultures (not filtered) and spiked samples (named “pre”, as they are observed before TTF) and samples after TTF (named “post”). Moreover, the presence of bacteria in filtrate samples was verified. NA is for Not Available data.

**Table S10**

| Kit for DNA extraction | Type of DNA extraction | cost/sample | Time of manipulation | Total time required |
| --- | --- | --- | --- | --- |
| Instagene Matrix (Bio-Rad) | one-step | About 1 € | 10’ | 1h 30’ |
| NucliSens Easymag (Biomerieux) | automated | About 15 € | 10’ | 2 h |

Table S10. Cost/sample and time required for both one-step and automate DNA extraction, disposable excluded. Prices are approximate.

**References**

Brakstad, Odd G., Kjetill Aasbakk, and Johan A. Maeland. "Detection of Staphylococcus aureus by polymerase chain reaction amplification of the nuc gene." *Journal of Clinical Microbiology* 30.7 (1992): 1654-1660.

Calvó, L., Martínez-Planells, A., Pardos-Bosch, J. and Garcia-Gil, L.J., 2008. A new real-time PCR assay for the specific detection of Salmonella spp. targeting the bipA gene. *Food Analytical Methods*, *1*(4), pp.236-242.

Devos, L., Clymans, K., Boon, N., & Verstraete, W. (2005). Evaluation of nested PCR assays for the detection of Legionella pneumophila in a wide range of aquatic samples. *Journal of applied microbiology*, *99*(4), 916-925.

He, Jian-Wen, and Sunny Jiang. "Quantification of enterococci and human adenoviruses in environmental samples by real-time PCR." *Applied and Environmental Microbiology* 71.5 (2005): 2250-2255.

Herlemann, D.P., Labrenz, M., Jürgens, K., Bertilsson, S., Waniek, J.J. and Andersson, A.F., 2011. Transitions in bacterial communities along the 2000 km salinity gradient of the Baltic Sea. *The ISME journal*, *5*(10), pp.1571-1579.

Huijsdens, X. W., Linskens, R. K., Mak, M., Meuwissen, S. G., Vandenbroucke-Grauls, C. M., & Savelkoul, P. H. (2002). Quantification of bacteria adherent to gastrointestinal mucosa by real-time PCR. *Journal of Clinical Microbiology*, *40*(12), 4423-4427.

Malinen, E., Rinttilä, T., Kajander, K., Mättö, J., Kassinen, A., Krogius, L., Saarela, M., Korpela, R. and Palva, A., 2005. Analysis of the fecal microbiota of irritable bowel syndrome patients and healthy controls with real-time PCR.*The American journal of gastroenterology*, *100*(2), pp.373-382.

Shannon, K. E., Lee, D. Y., Trevors, J. T., & Beaudette, L. A. Application of real-time quantitative PCR for the detection of selected bacterial pathogens during municipal wastewater treatment. *Science of the Total Environment*, *382*(1) (2007)., 121-129.

Wong, Yeng Pooi, Kek Heng Chua, and Kwai Lin Thong. "One-step species-specific high resolution melting analysis for nosocomial bacteria detection." *Journal of microbiological methods* 107 (2014): 133-137.

Zhang, R., Daroczy, K., Xiao, B., Yu, L., Chen, R., & Liao, Q. (2012). Qualitative and semiquantitative analysis of Lactobacillus species in the vaginas of healthy fertile and postmenopausal Chinese women. *Journal of medical microbiology*, *61*(Pt 5), 729-739.
